# Importance of Smell Loss to Patients With Chronic Rhinosinusitis With Nasal Polyps: Options for Management and Recovery

**DOI:** 10.1002/clt2.70149

**Published:** 2026-01-31

**Authors:** Thomas S. Higgins, Jennifer E. Douglas, Robert C. Kern, James N. Palmer, Sietze Reitsma, Martin Wagenmann, Rhea Goodman, Mark Corbett, Cristina Almansa, Amr Radwan

**Affiliations:** ^1^ Kentuckiana ENT A Division of ENT Care Centers Louisville Kentucky USA; ^2^ Department of Otolaryngology‐Head and Neck Surgery University of Louisville School of Medicine Louisville Kentucky USA; ^3^ Department of Otorhinolaryngology‐Head & Neck Surgery Hospital of the University of Pennsylvania Philadelphia Pennsylvania USA; ^4^ Department of Otorhinolaryngology, Head and Neck Surgery Northwestern University Feinberg School of Medicine Chicago Illinois USA; ^5^ Department of Otorhinolaryngology and Head and Neck Surgery Amsterdam University Medical Centre University of Amsterdam Amsterdam the Netherlands; ^6^ Department of Otorhinolaryngology Düsseldorf University Hospital (UKD) Düsseldorf Germany; ^7^ Regeneron Pharmaceuticals Inc. Tarrytown New York USA; ^8^ Sanofi Morristown New Jersey USA; ^9^ Sanofi Cambridge Massachusetts USA; ^10^ Regeneron Pharmaceuticals Inc. Uxbridge UK

**Keywords:** biologics, chronic rhinosinusitis with nasal polyps, olfaction, sinus surgery, smell loss

## Abstract

Primary diffuse type 2‐dominant chronic rhinosinusitis with nasal polyps (CRSwNP) is an inflammatory disease of the nasal cavity and paranasal sinuses associated with significant morbidity. Impaired sense of smell is a cardinal symptom of CRSwNP and one of the most burdensome for patients, impacting quality of life, mental health, and even safety. Mechanisms of smell loss in CRSwNP may be related to conductive losses due to significant burden of nasal polyps, as well as the impact of type 2 inflammatory mediators on olfactory sensory neurons. Initial medical management frequently involves intranasal or oral corticosteroids. Patients whose symptoms remain uncontrolled by medical treatment may be offered sinonasal surgery; however, patients may experience recurrence of smell loss following surgery. Guidelines recommend biologics for certain patients with CRSwNP after undergoing complete endoscopic sinus surgery, and data from clinical trials and real‐world evidence support their effectiveness in improving sense of smell. Given the impact of smell loss, shared decision‐making is important in identifying treatment options best suited to achieving patient goals. This review provides an overview of the importance of smell loss in CRSwNP and its known mechanisms, and reviews the evidence for the efficacy of current treatment options in restoring sense of smell.

## Plain Language Summary

Chronic rhinosinusitis with nasal polyps (CRSwNP) is a long‐term inflammatory disease of the nose and sinuses. In CRSwNP, small growths called polyps form inside the nasal passages. Symptoms include blocked and runny nose, loss of sense of smell, and facial pain or pressure. Loss of sense of smell is one of the worst symptoms for patients, and it affects their safety, mental health, and quality of life. The way that sense of smell is affected by CRSwNP is not fully understood, but there appears to be both a conductive component as well as a “type 2” immune component. Patients with loss of smell and CRSwNP are usually treated with steroids that can be applied locally in the nose or taken as a tablet. If steroids can’t control the symptoms, patients can have surgery to remove the polyps. Surgery can help, but many patients lose their sense of smell again. For some patients with CRSwNP, medical guidelines recommend biologic drugs (“biologics”) after complete sinus surgery, which can improve sense of smell. It’s important that healthcare providers work together with patients to understand what they want to achieve with their treatment, and to help find the right treatment for them.

## 1 Introduction

Reduced sense of smell is one of the cardinal symptoms of primary diffuse type 2‐dominant chronic rhinosinusitis with nasal polyps (CRSwNP) but may be underrated by the patient and/or the treating physician [1, 2]. Given the negative impact that loss of smell can have on overall morbidity, quality of life (QoL), patient safety, and patient nutrition, [3] a greater appreciation of the underlying mechanisms and potential treatments for this dimension of CRSwNP is needed.

This narrative review aims to provide an update on the potential mechanisms of smell loss in CRSwNP and an overview of the efficacy of the available treatment options in promoting recovery of sense of smell in patients with CRSwNP.

## 2 Importance of Smell Loss in CRSwNP

A systematic review from 2017 reported that 67%–78% of persons with chronic rhinosinusitis (CRS) experience smell impairment, and that the presence of nasal polyps was one of the most important factors associated with smell loss [4]. Patients with CRSwNP and lower airway comorbidities such as asthma and non‐steroidal anti‐inflammatory drug–exacerbated respiratory disease (NSAID‐ERD) tend to be the most affected [5, 6].

Patients and clinicians consider loss of smell to be one of the most important and most burdensome symptoms of CRSwNP [7, 8, 9]. A survey of patients with CRSwNP found that 71% rated impaired sense of smell as their most debilitating symptom, and that effective treatment of their smell and taste symptoms contributed to their overall satisfaction with CRSwNP treatment [7]. Further, a survey of otorhinolaryngologists revealed that 80% considered impaired sense of smell as one of the most important symptoms in defining uncontrolled CRSwNP [9].

Although patients with CRS frequently have olfactory dysfunction, it can be difficult to delineate the contribution of CRS itself versus the impact of other potential underlying factors such as diabetes, advanced age, asthma, or smoking [10, 11].

Data from randomized clinical trials of biologics for CRSwNP further demonstrate the high prevalence and severity of loss of smell among patients with the most severe forms of CRSwNP, who in many cases have previously required sinus surgery. [12, 13, 14] In patients in the SINUS‐24 and SINUS‐52 pivotal clinical trials for dupilumab, 87% of patients rated “decreased sense of smell/taste” as one of the most important items in the 22‐item Sino‐Nasal Outcome Test (SNOT‐22), with 82% stating that the problem was “severe” or “as bad as can be.” [8] Findings were similar in the mepolizumab pivotal trial, SYNAPSE, in which 80% (placebo arm) and 78% (mepolizumab arm) of patients reported their decreased sense of smell/taste to be “as bad as it can be.” [15] Patients in the aforementioned clinical trials, in fact, were commonly anosmic at baseline (Table 1), as evidenced by the severity of dedicated symptom and psychophysical scores, such as the University of Pennsylvania Smell Identification Test (UPSIT) [12, 13, 14, 15].

**TABLE 1:** Non‐comparative cross‐trial summary of baseline loss of smell and improvement in sense of smell with biologic treatment in patients with CRSwNP in phase 3 clinical trials.

	Psychophysical measures		Symptom scores	
	Placebo	Biologic	Placebo	Biologic
**SINUS‐24** [12]**: Placebo (*n* = 133) versus dupilumab q2w (*n* = 143)**	**UPSIT (range 0–40)**		**LoS (range 0–3)**	
Baseline, mean (SD)	14.44 (8.31)	14.68 (8.66)	2.73 (0.51)	2.70 (0.57)
Wk 24, mean (SD)	14.56 (8.58)	25.39 (9.49)	2.50 (0.77)	1.35 (0.99)
Change from baseline at Wk 24, LS mean (SD)	0.70 (0.71)	11.26 (0.67)	−0.29 (0.07)	−1.41 (0.07)
Treatment difference (95% CI)	10.56 (8.79, 12.34) *p* < 0.0001		−1.12 (−1.31, −0.93) *p* < 0.0001	

**SINUS‐52** [12]**: Placebo (*n* = 153) versus dupilumab (*n* = 150** ^a^ **)**	**UPSIT (range 0–40)**		**LoS (range 0–3)**	
Baseline, mean (SD)	13.78 (8.31)	13.46 (8.20)^a^	2.72 (0.52)	2.81 (0.46)^a^
Wk 24, mean (SD)	13.30 (7.96)	23.89 (9.21)^b^	2.49 (0.79)	1.55 (1.02)^b^
Change from baseline at Wk 24, LS mean (SE)	−0.81 (0.71)	9.71 (0.56)^b^	−0.23 (0.08)	−1.21 (0.06)^b^
Treatment difference (95% CI)	10.52 (8.98, 12.07)^b^ *p* < 0.0001		−0.98 (−1.15, −0.81)^b^ *p* < 0.0001	

**SYNAPSE** [13, 15]**: Placebo (*n* = 201) versus mepolizumab (*n* = 206)**	**UPSIT (range 0–40)**		**Loss of smell VAS (range 0–10)**	
Baseline, mean (SD)	13.4 (7.45)	13.0 (6.81)	9.7 (0.60)	9.6 (0.83)
Change from baseline at Wk 52, mean (SD) unless otherwise stated	0.4 (8.6)^c^	1.7 (10.8)^c^	Change at Wks 49–52: −1.4 (2.65)	Change at Wks 49–52: −2.8 (3.61)
Treatment difference (95% CI)	Adjusted difference in medians: 0.40 (−1.49, 2.28) *p* = 0.30		−0.37 (−0.65, −0.08) *p* = 0.020	

**POLYP‐1** [14]**: Placebo (*n* = 66) versus omalizumab (*n* = 72)**	**UPSIT (range 0–40)**		**LoS (range 0–3)**	
Baseline, mean (SD)	13.9 (7.4)	12.8 (7.9)	2.8 (0.4)	2.5 (0.8)
Change from baseline at Wk 24, adjusted mean (SE)	0.63 (0.90)	4.44 (0.84)	−0.23 (0.10)	−0.56 (0.09)
Treatment difference (95% CI)	3.81 (1.38, 6.24) *p* = 0.0024		−0.33 (−0.60, −0.06) *p =* 0.0161	

**POLYP‐2** [14]**: Placebo (n = 65) versus omalizumab (n = 62)**	**UPSIT (range 0–40)**		**LoS (range 0–3)**	
Baseline, mean (SD)	13.1 (7.3)	12.8 (7.6)	2.8 (0.6)	2.6 (0.8)
Change from baseline at Wk 24^b^	0.44 (0.81)	4.31 (0.83)	−0.13 (0.10)	−0.58 (0.10)
Treatment difference (95% CI)	3.86 (1.57, 6.15) *p* = 0.0011		−0.45 (−0.73, −0.16) *p =* 0.0024	

**OSTRO** [16]**: Placebo (*n* = 203) versus benralizumab (*n* = 207)**	**UPSIT (range 0–40)**		**DSS score (range 0–3)**	
Baseline, mean (SD)	NR	NR	NR	NR
Change from baseline at Wk 56, LS mean	NR	NR	−0.187^d^	−0.423^e^
Treatment difference (95% CI)	Data NR but no treatment difference		−0.237 (−0.389, −0.084) *p* = 0.002	

**WAYPOINT** [17]**: Placebo (*n* = 205) versus tezepelumab (*n* = 203)**	**UPSIT (range 0–40)**		**LoS (range 0–3)**	
Baseline, mean (SD)	11.9 (5.9)	13.1 (7.3)	2.8 (0.4)	2.9 (0.4)
Wk 52, mean (SD)	14.7 (7.89)	22.6 (8.77)	2.43 (0.83)	1.55 (1.07)
Change from baseline at Wk 52, LS mean (SE)	−0.15 (0.60)	9.31 (0.60)	−0.26 (0.06)	−1.26 (0.06)
Treatment difference (95% CI)	9.46 (7.82, 11.10) NR		−1.00 (−1.18, −0.83) *p* < 0.001	

**ANCHOR‐1 and ‐2 (integrated data shown)** [18]**: Placebo (*n* = 256) versus depemokimab (*n* = 272)**	**UPSIT (range 0–40)**		**LoS (range 0–3)**	
Baseline, mean (SD)	NR	NR	2.76 (0.50)	2.77 (0.48)
Change from baseline at Wks 49–52, LS mean (SE)	NR	NR	−0.3 (0.049)^f^	−0.52 (0.048)^g^
Treatment difference (95% CI)	NR		−0.22 (−0.35, −0.08) *p* = 0.002	

Abbreviations: CI, confidence interval; CRSwNP, chronic rhinosinusitis with nasal polyps; DSS, difficulty in sense of smell; LoS, loss of smell; LS, least squares; NR, not reported; q2w, every 2 weeks; q4w, every 4 weeks; SD, standard deviation; SE, standard error; SNOT‐22, 22‐item Sino‐Nasal Outcome Test; UPSIT, University of Pennsylvania Smell Identification Test; VAS, visual analog scale; Wk, Week.

^a^ q2w arm, *n* = 150.

^b^ Pooled q2w arm and q2w–q4w arm before switch to q4w, *n* = 295.

^c^ *n* = 54.

^d^ *n* = 175.

^e^ *n* = 179.

^f^ *n* = 227.

^g^ *n* = 244.

A number of studies suggest that impaired sense of smell in CRS is correlated with objective or physician‐reported measures of disease severity. For example, in the SINUS‐24 and SINUS‐52 clinical trials, change in loss of smell (LoS), SNOT‐22 smell/taste item, and UPSIT scores with dupilumab treatment (*n* = 438) were found to correlate with change in Lund–Mackay computed tomography scores [19]. A prospective study of 148 patients also demonstrated significant correlation between Sniffin’ Sticks threshold, discrimination, and identification score and opacification of the olfactory cleft [20]. A cross‐sectional study of 34 patients and a prospective case control study of 67 patients with CRS found that olfactory scores (Sniffin’ Sticks and 40‐item Smell Identification Test [SIT40], respectively) were also associated with levels of inflammatory cytokines in the olfactory cleft [21, 22]. Further, a cross‐sectional analysis of 367 patients with CRS demonstrated that olfactory scores moderately correlated with endoscopy scores [20]. This was supported by data from the SINUS‐24 and SINUS‐52 trials, in which patients with anosmia as measured by UPSIT had significantly higher nasal polyp score (NPS) than patients without anosmia, with UPSIT and LoS scores both found to correlate with NPS [23]. However, in a meta‐analysis of summary statistics from 5 studies on SIT40 and 3 studies on Sniffin’ Sticks, correlations with endoscopy scores were not detectable [24]. Impaired sense of smell is also known to exert a negative effect on patients' overall and health‐related QoL [3, 25, 26]. Many patients with olfactory disorders report decreased enjoyment of food, changes in appetite and food intake, and safety concerns around failure to smell risks such as fire, gas, or spoiled food. Patients with smell disorders also report difficulty with personal hygiene, in their social lives, and the negative impact on mood and mental health [3, 7].

In a prospective survey of patients with CRS, 23% had impaired eating‐related QoL, including reduced enjoyment of food, eating less, or having difficulty with weight [27]. A second survey of patients with CRSwNP found that 84% reported frequent impact of smell loss on their enjoyment of food and drink; 75% and 74% reported frequent impact on food safety and gas or smoke safety, respectively; 66% and 60% reported frequent impact on their personal hygiene and the hygiene of their children or pets, respectively; and 63% and 39% reported frequent impact on their mood and relationships, respectively [7]. Further, in patients with CRSwNP, a retrospective observational study found that patients with CRSwNP and anosmia had worse scores on measures of anxiety, depression, and phobia than those without anosmia [26].

Despite the wide‐ranging impact of smell loss, the psychophysical testing of smell is not commonly performed by physicians in the clinic due to a number of factors, but most commonly due to a lack of insurance reimbursement and a scarcity of time during scheduled patient encounters. Consequently, both patients and medical professionals may not recognize the impact of smell loss with regard to its effect on patient QoL, suggesting a need for improved education and understanding [1, 28].

## 3 Potential Mechanisms of Smell Loss in CRSwNP

Within the overall CRSwNP phenotype, different endotypes (which can be associated with type 1, type 2, or type 3 molecular biomarkers) or a combination thereof, [29, 30] may have varying impacts on olfaction [30, 31]. In most Western regions, and increasingly in other parts of world, CRSwNP displays a predominantly type 2 inflammatory signature [30, 31, 32, 33]. Biomarker studies in patients with CRS and CRSwNP found that smell loss was significantly associated with the presence of a type 2 inflammatory endotype, but not with type 1 or type 3 endotypes [30, 34].

The mechanism behind smell loss in CRSwNP is not fully elucidated, and is likely multifactorial, potentially influenced by direct effects of type 2 inflammatory mediators on the number and function of olfactory sensory neurons, as well as possible loss of conduction due to nasal polyp burden [35, 36, 37]. Other possible mechanisms of smell loss include upper respiratory tract viral infections, head trauma, neurodegeneration, and aging [35]. Multiple studies have shown that the extent and location of nasal polyps either does not or only weakly correlates with degree of smell loss [23, 24, 38, 39, 40]. The data remain mixed, however, with some evidence of the differential association of olfactory cleft opacification with smell loss based on nasal polyp status in CRS [20].

**FIGURE 1:**
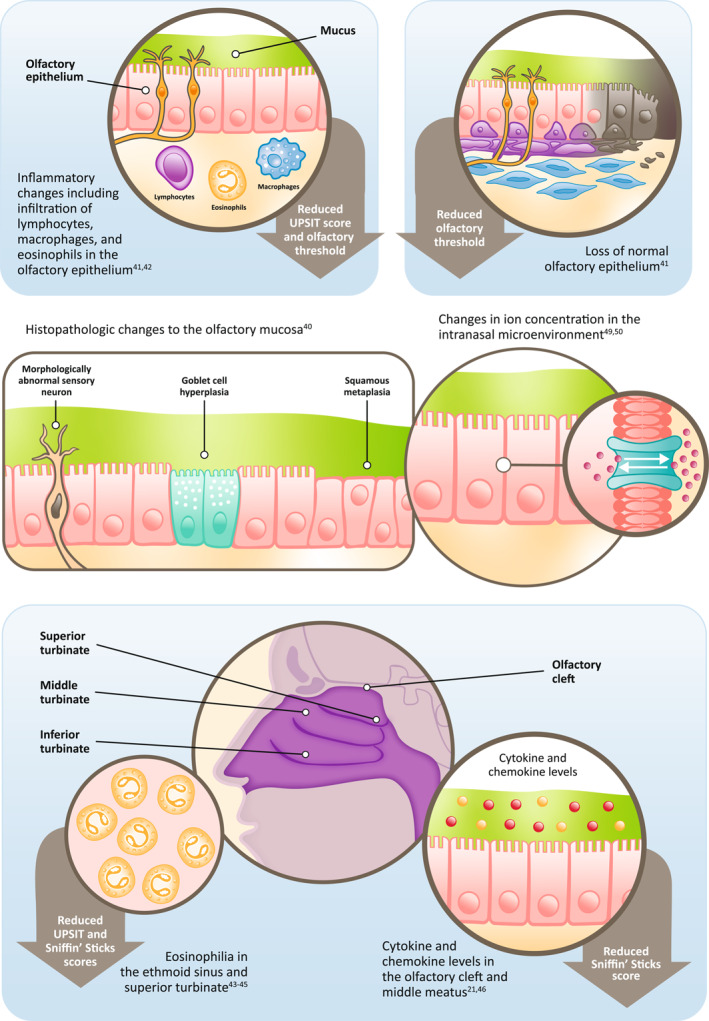
Evidence for mechanisms of smell loss in CRSwNP. CRSwNP, chronic rhinosinusitis with nasal polyps; UPSIT, University of Pennsylvania Smell Identification Test.

There are several other lines of evidence regarding the possible mechanisms of smell loss in CRSwNP [35, 37, 41] (Figure 1). In patients with CRS, histologic examination of the olfactory mucosa has shown goblet cell hyperplasia, squamous metaplasia, and morphologic abnormalities in olfactory sensory neurons compared with controls [42]. Patients with greater smell impairment displayed a loss of normal olfactory epithelium and were more likely to have moderate‐to‐severe inflammatory changes in the olfactory mucosa, including infiltration of lymphocytes, macrophages, and eosinophils [43, 44]. Further, eosinophilia in the ethmoid sinuses and superior turbinate in patients with CRSwNP has also been associated with greater smell impairment [45, 46, 47]. A number of inflammatory cytokines have also been linked to smell impairment [21, 22, 48, 49, 50]. A study assessing the role of cytokines in olfactory dysfunction found that levels of interleukin (IL)‐5 in the olfactory cleft mucus were significantly inversely correlated with smell threshold, discrimination, and identification (TDI) scores in both patients with CRSwNP and patients with CRS without nasal polyps (CRSsNP). In the CRSwNP patient population, levels of IL‐6, IL‐7, and vascular endothelial growth factor A were also significantly correlated with TDI score [21]. These data are supported by more recent work in patients with CRS, confirming an association between olfaction and mucus levels of a range of cytokines, including IL‐2, IL‐5, IL‐6, IL‐10, and IL‐13 in the olfactory cleft, and IL‐2, IL‐5, and IL‐13 in the middle meatus [22, 48, 49]. Moreover, in an endotyping cluster analysis of patients with CRS, those with the lowest olfactory scores were found to be in clusters dominated by elevated levels of IL‐5, IL‐13, and immunoglobulin E (IgE) [50].

The exact mechanisms of how type 2 inflammatory cytokines impact the olfactory pathways are still unknown. Mouse models suggest a possible role for type 2 inflammatory mediators in regulation of olfactory stem cell activation [51, 52, 53]. Moreover, IL‐4 and IL‐13 have been shown to have non‐redundant roles in olfactory function [54]. In a mouse model, IL‐4, but not IL‐13, signaling had a direct role in impairing function of olfactory sensory neurons, eliciting inflammatory responses in the olfactory epithelium, and switching on a neuroinflammatory microenvironment with neuronal crosstalk with mast cells, macrophages, and natural killer cells [54].

There is evidence in healthy controls to suggest that changes in ion concentrations in the intranasal microenvironment can modulate olfactory performance [55]. Supporting this, chloride secretion in the nasal epithelia has been shown to be reduced in patients with CRSwNP both in vivo and in primary nasal epithelial cultures [56]. In the latter, IL‐13 expression was found to be 6‐fold higher than in control cultures and impacted the regulation of chloride secretion. The rapid smell restoration observed with dupilumab or steroids [57, 58] suggests that factors other than polyp shrinkage or stem cell regeneration may be involved in the recovery of olfaction, such as reduced type 2 inflammatory signaling and/or changes in ion concentrations.

## 4 Assessment of Sense of Smell

Reduction or loss of smell is one of the core symptoms of CRSwNP as defined in the European Position Paper on Rhinosinusitis and Nasal Polyps 2020 [2]. However, while it is recommended that physicians ask patients about reduced sense of smell, this may not always be assessed as part of the standard evaluation, where the focus may be on the more apparent symptoms of nasal congestion, rhinorrhea/postnasal drip, and facial pain/pressure [59].

A number of smell assessment tools are available for everyday clinical practice, such as the visual analog scale (VAS), smell tests, and QoL questionnaires. For more detailed assessment of smell loss, olfactory psychophysical tests may be appropriate. These tests can be specific for the region/country where they have been developed and validated to account for the cultural dependence of odor identification tasks [36]. In the United States, the most commonly used smell identification test is UPSIT, whereas in Europe, the Sniffin’ Sticks test is more often used [36]. The recently developed SCENTinel smell test, which allows for faster and more affordable testing, is also beginning to be used [60].

**FIGURE 2:**
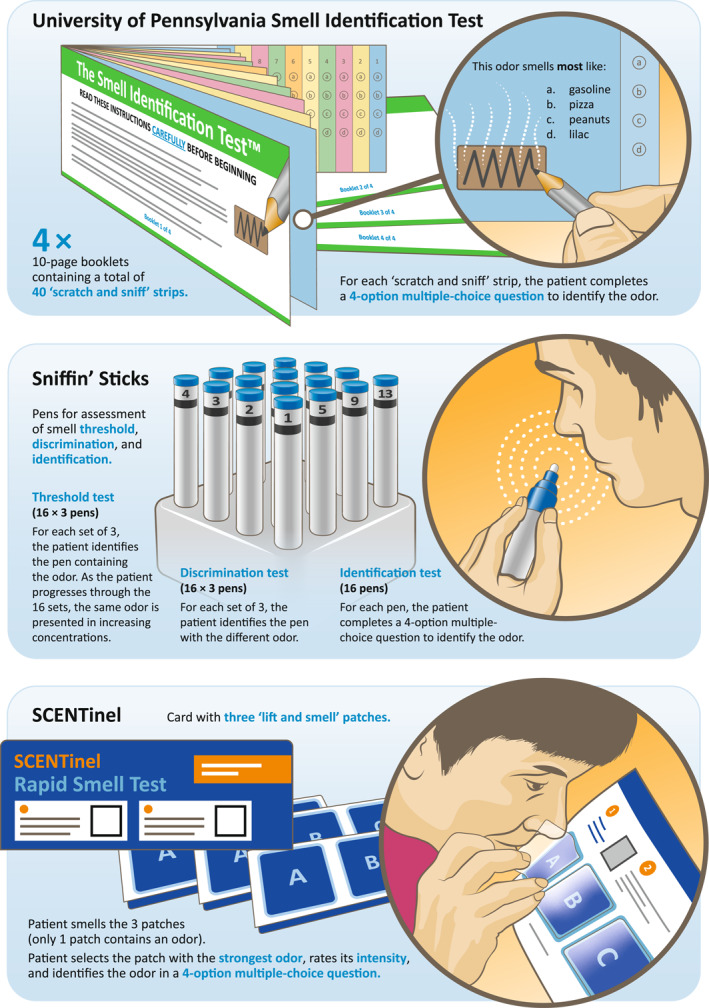
Commonly used psychophysical smell assessments.

Figure 2 provides an overview of the commonly used psychophysical smell tests, and Figure S1 outlines their thresholds for identifying degrees of smell loss. UPSIT (range 0–40) defines anosmia as a score of ≤ 18, and hyposmia can be defined as severe (19–25), moderate (26–29 in males; 26–30 in females), or mild (30–33 in males; 31–34 in females) [61]. The Sniffin’ Stick test (range 1–48) combines scores from threshold (1–16), discrimination, and identification tests (each 0–16) to give an overall TDI score [62]. Anosmia is defined as a TDI score of ≤ 16.5, and hyposmia as a score of 16.5–30.5 [63]. In the SCENTinel smell test, patients are assessed on their pattern and accuracy of response to the following tests: odor detection (correct/incorrect), odor intensity (rating of ≤ 20 or ≥ 21 on a scale of 0–100), and odor identification (correct/incorrect). An overall score is created by weighing the contribution of each smell function [60, 64].

A recent systematic review examining the relationship between outcome measures in sinonasal disorders found that olfactory dysfunction as measured by the Sniffin’ Stick test correlated strongly (*r* > 0.7) with patient‐reported outcomes, including VAS for smell and SNOT‐22 total score in patients with CRS, while UPSIT typically displayed weak correlations (*r* < 0.35) [65]. The weak correlation of UPSIT with patient‐reported outcomes may be due, in part, to the fact that UPSIT assesses odor identification alone and does not account for impairment in olfactory threshold [65]. Opacification of the olfactory cleft, visible on CT scan, correlates with olfaction as determined by psychophysical testing in patients with CRSwNP, [20] though the correlation of Lund–Mackay CT score with simple smell measures such as the LoS score and SNOT‐22 smell/taste item was found to be weak [19].

## 5 Management of Smell Loss in CRSwNP

As loss of smell is considered one of the most burdensome symptoms for patients with CRSwNP, [8, 36] recovery of smell can be considered a useful indicator of successful treatment. Primary medical treatment of CRSwNP involves intranasal corticosteroids, which can be administered through a number of different methods, including nasal irrigation, drops, sprays, or exhaled delivery systems. As a second‐line treatment, patients may also receive short bursts of oral corticosteroids (OCS). If these treatments fail to provide lasting control of symptoms, sinonasal surgery is an option for some patients. Data on the indicative impact of each intervention on smell recovery are collected in Table 2 [66, 67, 68]. In patients whose disease remains uncontrolled, biologic therapy is indicated for those with bilateral nasal polyps despite prior surgery who meet at least 3 of the following criteria: evidence of type 2 inflammation, need for two or more courses of systemic corticosteroids within the last year, significantly impaired QoL, significant loss of smell, or coexisting asthma. [2]

**TABLE 2:** Indicative impact of medical and surgical interventions on olfaction in patients with CRSwNP.

	Intranasal corticosteroids [66]				Oral corticosteroids [67]	Surgery [68]
	UPSIT (range 0–40) Mean difference (95% CI) versus baseline				BAST‐24 (range 0–100) Mean (SD), *p* value versus baseline	UPSIT (range 0–40) Mean difference (95% CI) versus baseline
	Spray	Rinse	Drops	EDS		
Treatment effect	3.24 (2.05, 4.42)	2.77 (–0.84, 6.39)	5.03 (1.89, 8.18)	4.10 (1.69, 6.52)	Baseline: Smell detection: 30.7 (39.5) Smell identification: 7.1 (16.1) Forced choice: 13.8 (23.3) Week 2: Smell detection: 60.9 (42.8), *p* < 0.05 Smell identification: 20.4 (22.8), *p* < 0.05 Forced choice: 31.1 (30.8), *p* < 0.05	7.87 (2.25, 13.49)

*Note:* Data from the referenced meta‐analyses. Baseline scores and follow‐up times were not specified.

Abbreviations: BAST‐24, Barcelona smell test‐24; CI, confidence interval; CRSwNP, chronic rhinosinusitis with nasal polyps; EDS, exhalation delivery system; UPSIT, University of Pennsylvania Smell Identification Test.

Given the risk of adverse effects with OCS, and recognizing the need for shared decision‐making, the classical stepwise approach—topical corticosteroids, then OCS, then surgery and/or biologics—is increasingly being questioned, and individualized approaches are being encouraged [69, 70]. Research into the association of clinical presentation of CRS with disease endotypes may have the potential to provide valuable insight into the underlying mechanism of disease for individual patients [30]. In the future, this may prove beneficial in understanding which treatments may be best suited to the patient.

### 5.1 Olfactory Training

Olfactory training has been shown to provide benefit for patients across a range of underlying etiologies of smell loss [71, 72]. Specifically in patients with CRS, those who underwent 12 weeks of olfactory training following sinonasal surgery were more likely to achieve clinically relevant improvements in olfactory function as measured by the Sniffin’ Stick test [73]. Similarly, in patients with CRS following nasal endoscopy, early olfactory training resulted in significantly improved odor detection and recognition, and improved QoL measures, compared with those without olfactory training [74]. Overall, however, evidence for olfactory training in CRSwNP is limited, and the International Consensus Statement on Allergy and Rhinology recommends olfactory training only for patients with olfactory dysfunction with no underlying disease [71].

### 5.2 Medical Management

A 2016 review by Gudis and Soler examined the efficacy of standard CRSwNP therapies with regard to smell recovery [75]. Overall, short term OCS (< 3 weeks) demonstrated efficacy in the treatment of CRS‐associated smell loss for both subjective and objective outcome measures. The long‐term efficacy of OCS remains unclear; however, systematic evaluation of their long‐term use is unfeasible due to significant side effects. Indeed, studies have shown that even short bursts of corticosteroids are associated with increased risk of adverse events such as sepsis and bone fractures, [76] and should, therefore, be prescribed sparingly. In general, the evidence supporting topical corticosteroids alone in the treatment of CRS‐associated olfactory dysfunction is less compelling than the evidence for OCS. However, several studies have demonstrated subjective olfactory improvement and the low incidence of systemic side effects, making topical corticosteroids a feasible long‐term treatment option [75].

The method of delivery for topical corticosteroids can impact the distribution of treatment to the sinuses. As such, high‐volume devices are recommended over low‐volume devices such as nasal drops or sprays [77]. In comparison to nasal sprays, exhaled delivery systems have been shown to deposit corticosteroids throughout the nasal cavity [78]. In the NAVIGATE I study of patients with CRSwNP, the exhalation delivery system with fluticasone (EDS‐FLU) significantly improved measures of smell as early as Week 4 in patients who had previously remained symptomatic despite prior corticosteroid use and/or surgery [79]. Further studies also support the efficacy of EDS‐FLU in reducing CRS symptoms and exacerbations [80]. Steroid irrigation is also known to significantly improve distribution of treatment in the sinuses [81]. A randomized controlled trial of patients with olfactory loss demonstrated that significantly higher proportions of patients receiving olfactory training achieved improvements in olfaction with steroid irrigation compared with those receiving saline irrigation [82]. Intranasal budesonide has also been shown to maintain improvements in sense of smell in patients with CRSwNP and asthma following 2 weeks of OCS [83]. However, while topical corticosteroids delivered to the olfactory cleft have clear value as a first‐line treatment for smell loss in patients with CRSwNP, recent reviews note the impact of variable treatment protocols and the relatively limited data for use of steroid irrigation [84, 85]. As such, effective methods of administration for topical corticosteroids to the olfactory cleft should be a focus for clinicians.

### 5.3 Surgery

Numerous studies have examined the effect of sinus surgery on olfactory outcomes, with mixed findings, which may be exacerbated by heterogeneity in patient populations and surgical procedures/techniques [86]. However, several studies provide evidence to support the use of endoscopic sinus surgery (ESS) in the treatment of olfactory dysfunction in CRS.

Surgery has been shown to significantly improve multiple measures of olfaction as early as 2 weeks post‐surgery, including olfactory threshold test, sense of smell scores, and “experience of smell and taste” scores [87]. However, a systematic review, which comprised 24 studies and nearly 2000 patients with CRS, found that sense of smell improved in only approximately half of patients after surgery [88]. Patients who showed improvement in sense of smell after surgery generally maintained the improvements in the short‐to‐medium term (follow‐up 0.5–18 months), though a separate analysis found that smell loss symptoms can recur at around 3 months post‐surgery [89].

The extent of nasal polyp load has been shown to influence the chance of post‐ESS olfaction recovery [90]. Further, the extent of surgery has been reported to influence improvement in olfaction in patients with CRS, with those undergoing complete sinus surgery—defined as bilateral maxillary antrostomies, bilateral total ethmoidectomies, bilateral sphenoidotomies, or bilateral frontal sinusotomies—achieving significantly greater improvements in the Brief Smell Identification Test compared with those undergoing targeted surgical intervention [91]. Supporting this, patients undergoing “radical” ESS were found to have greater improvement in olfaction at 1 year follow‐up compared with those undergoing “functional” ESS [92]. However, some studies suggest that more extensive surgery may impact response to future treatment, or may result in less improvement in QoL. In a retrospective analysis of 145 patients with prior ESS receiving dupilumab for CRSwNP, more extensive ESS was associated with worse olfactory outcomes compared with less extensive ESS [93]. Further, a retrospective analysis of 247 patients with CRS who underwent ESS at a single center found that patients whose surgery was more extensive than indicated by preoperative imaging had less improvement in SNOT‐22 after 2 years than patients whose extent of surgery was concordant with preoperative imaging. However, patients with more extensive surgery in this study were significantly less likely to have nasal polyposis, which may limit the generalizability of these findings [94]. The use of postoperative topical corticosteroids may also influence smell outcomes following ESS, with 1 prospective cohort study demonstrating sustained improvement in sense of smell for up to 6 months with the addition of daily fluticasone furoate. Further, the use of postoperative topical corticosteroids has been shown to improve smell to a similar degree as postoperative systemic corticosteroids (SCS), [95] with improvements maintained up to 2 years. Administration of postoperative topical corticosteroids via steroid‐eluting bioabsorbable devices has also been shown to maintain long‐term symptom improvement with reduced need for SCS [96].

Unfortunately, evidence on improvement in sense of smell after surgery remains non‐concordant, likely due to variability in the extent of surgery and in the administration of corticosteroids in the olfactory cleft in the prolonged postoperative period. While 1 analysis revealed that ESS improved the likelihood of achieving smell recovery compared with continued medical therapy, [97] another found that ESS had little impact on smell recovery [98]. Further, a 2014 Cochrane review comparing surgical and medical therapies for CRSwNP found little difference in terms of impact on patient‐reported symptom scores and QoL outcomes at 12 months, though the quality of evidence was rated as “low” or “very low” and some of the treatment regimens in the trials comprising this review no longer meet current recommended standards [99]. For example, postoperative steroid irrigation, included in this review, only came into common use after 2014. While literature around the impact of ESS on objective measures including UPSIT and Sniffin’ Sticks is limited with mixed findings, [98, 100, 101] a 2016 meta‐analysis concluded that ESS resulted in significant improvement in Sniffin’ Sticks scores in patients with CRSwNP, but not in a mixed cohort of patients with CRSwNP or CRSsNP [68]. Nevertheless, irrespective of the impact on olfaction, sinus surgery has value in facilitating the delivery of topical therapies, providing tissue sampling for accurate diagnosis, and managing CRSwNP complicated by extension to adjacent structures [102, 103].

### 5.4 Assessing Response to Medical or Surgical Treatment

Research into factors affecting olfactory response to corticosteroids is limited. However, a study by Bogdanov et al. found that patients who did not experience improvement in olfaction following corticosteroid treatment also did not benefit from surgery [104]. Through practical experience, the authors also found that patients who have subjective return of flavor with corticosteroids may be likely to benefit from surgery or dupilumab. Indeed, improvements in sense of “taste” and sense of smell in patients with CRSwNP treated with dupilumab have been shown to be moderately associated [105]. Further, a retrospective analysis of pre‐biologic OCS responsiveness found that patients who experienced smell improvement following OCS treatment were more likely to report smell recovery after treatment with dupilumab [106]. Conversely, a number of factors are known to affect recovery of sense of smell following surgery. Patients with CRSwNP with anosmia, with more severe polyposis, or with a higher ethmoid‐to‐maxillary opacification ratio before surgery had increased olfactory benefit from surgery [90, 107, 108]. Surgery‐naïve patients also had a higher chance of improvement in olfaction compared with those with prior surgery [88]. In particular, patients with 3 or more prior surgeries reported minimal improvement in patient‐reported outcomes, including VAS for impaired smell [109]. Indeed, some patients, including those with normal sense of smell, report worse olfactory dysfunction following ESS, [108] though it is not clear whether worsening sense of smell is a consequence of irreversible scarring from surgery or of neurogenic exhaustion owing to the chronicity of the disease [110, 111]. Longer duration of smell impairment and blood eosinophilia also adversely affected smell recovery following surgery [112].

## 6 Biologics and Smell Recovery

Biologics targeting type 2 inflammation are an emerging option for patients with uncontrolled CRSwNP [113, 114]. Efficacy with regard to smell improvement has been demonstrated in randomized clinical trials for biologics with a range of mechanisms of action, including dupilumab (inhibits IL‐4/IL‐13), [12, 23] mepolizumab, [13, 15] benralizumab, [16] and depemokimab [18] (all inhibit IL‐5), omalizumab (inhibits IgE), [14] and tezepelumab (inhibits thymic stromal lymphopoietin) [17] (Table 1). Dupilumab is known to be efficacious with regard to smell improvement irrespective of patients' surgical history, and in patients with other coexisting type 2 conditions including asthma, NSAID‐ERD, or allergic rhinitis [115, 116, 117, 118]. For mepolizumab, the SYNAPSE clinical trial exclusively recruited patients with a history of surgery and demonstrated significant improvements in smell measures versus placebo, though improvements were lesser in patients with ≥ 2 previous surgeries [13, 15, 119]. Mepolizumab also improves smell irrespective of coexisting asthma or NSAID‐ERD [120]. For omalizumab, subgroup analysis of the POLYP clinical trials demonstrated smell improvement in patients with or without prior surgery, asthma, or NSAID‐ERD [121]. Improvements in smell were observed with depemokimab and tezepelumab versus placebo in the ANCHOR (verbal response scale) and WAYPOINT (LoS score) studies, respectively [17, 18]. In the OSTRO study, smell improvements with benralizumab versus placebo were observed with the difficulty in sense of smell score but not by UPSIT [16]. With regard to the timescale of improvement, dupilumab rapidly and significantly improved sense of smell, with benefit seen by the third day of treatment [57]. Improvements in smell score with tezepelumab and depemokimab versus placebo were observed at the first post‐treatment assessment at week 2 and weeks one to four, respectively [17, 18]. There is currently no clinical trial evidence examining the rapidity of smell improvement for mepolizumab, omalizumab, or benralizumab. Data from clinical trials are supported by emerging real‐world evidence demonstrating improvements in smell with biologic therapy, including in patients with coexisting conditions such as asthma [122, 123, 124, 125, 126, 127, 128]. There is also preliminary evidence comparing the effectiveness of biologics and surgery, though results are mixed. For dupilumab, 2 studies found equal olfactory benefit when compared with ESS by 6 and 12 months for the respective studies, [129, 130] while for omalizumab and mepolizumab, 2 and 1 studies, respectively, found that ESS yielded greater olfactory improvements by 6 and 12 months, respectively [130, 131].

Overall, recent systematic reviews reported that dupilumab was more efficacious for improving sense of smell than omalizumab, mepolizumab, and benralizumab [132, 133, 134, 135]. Further, the recent EVEREST head‐to‐head, phase 4 trial of dupilumab versus omalizumab demonstrated that dupilumab was superior to omalizumab in improving sense of smell in patients with CRSwNP and coexisting asthma [136]. Improvement in sense of smell with dupilumab treatment was found to be independent of reduction in nasal polyp size, and improvement was observed in patients who underwent multiple surgeries, which may suggest that resolution of type 2 inflammation drives recovery of sense of smell [12, 23, 39].

## 7 Shared Decision‐Making in the Management of Smell Loss in CRSwNP

A recent patient advisory board statement from the European Forum for Research and Education in Allergy and Airway Diseases (EUFOREA) captured patients' frustration at the underestimation of CRSwNP disease burden, and detailed the need for greater physician awareness of the impact of smell loss on patient wellbeing [137]. Supporting the patient advisory board statement, a multicenter survey study highlighted the differences between patient‐ and physician‐reported symptom frequency and severity [138]. Further, a patient experience survey found that patients rated loss/reduced sense of smell as their most troublesome symptom, and that satisfaction with the management of smell loss was consistently lower than overall satisfaction with CRSwNP management [7].

A recent review on shared decision‐making in the management of CRSwNP notes that physicians should include discussion of a patient's symptoms, goals, and ease of treatment compliance alongside considerations around treatment efficacy, risk, and cost [69]. Given the difficulties that some patients face in finding treatments that are suitably effective, with manageable administration, treatment adherence, and side effects, [137, 139] discussion around switching treatment strategy may also be valuable [140].

Further research may enable a more personalized approach to the treatment of smell loss in the future. For example, research into biomarkers or endotypes of smell loss may support the identification of patients who may be less likely to respond to conventional treatments or more likely to respond to biologics. Understanding the impact of the extent and type of sinus surgery and how this correlates with sense of smell and inflammatory markers in the olfactory cleft may also enable surgery to be tailored to a greater extent to individual patient needs. Finally, understanding the impact of environmental factors, such as smoking or pollution, and of coexisting conditions, such as diabetes or neurodegenerative or mental health conditions, may enable a more holistic approach to the treatment of smell loss.

## 8 Conclusions

CRSwNP significantly affects patients' QoL, particularly through the impairment of the sense of smell, a symptom that is often overlooked in clinical practice. The loss of smell is linked to type 2 inflammation, which affects olfactory sensory neurons and contributes to the complexity of the condition. However, further human studies are needed to more clearly elucidate the molecular mechanisms of smell loss and the cytokines involved, including greater understanding of the role of type 1 and 3 inflammation, and the long‐term molecular and cellular impact of inflammation on olfactory sensory neurons. Current treatment strategies include corticosteroids, surgical options, and biologic therapies, all of which have demonstrated varying degrees of success in restoring olfactory function. However, many patients still experience recurrent smell loss post‐surgery. The importance of shared decision‐making in treatment planning is emphasized, as understanding patient preferences and treatment goals can lead to more tailored and effective management strategies. Additional studies into endotypes of smell loss, the impact of the extent and type of sinus surgery, and the role of environmental factors and coexisting conditions may also enable greater optimization and personalization of treatment options for patients with CRSwNP.

## Author Contributions

**Thomas S. Higgins:** conceptualization, writing – original draft, writing – reviewing and editing. **Jennifer E. Douglas:** conceptualization, writing – original draft, writing – review and editing. **Robert C. Kern:** conceptualization, writing – original draft, writing – review and editing. **James N. Palmer:** conceptualization, writing – original draft, writing – review and editing. **Sietze Reitsma:** conceptualization, writing – original draft, writing – review and editing. **Martin Wagenmann:** conceptualization, writing – original draft, writing – review and editing. **Rhea Goodman:** supervision, conceptualization, writing – original draft, writing – review and editing. **Mark Corbett:** conceptualization, writing – original draft, writing – reviewing and editing. **Cristina Almansa:** writing – reviewing and editing. **Amr Radwan:** conceptualization, writing – original draft, writing – review and editing.

## Funding

Funding support was provided by Regeneron Pharmaceuticals Inc. and Sanofi in accordance with the Good Publication Practice guidelines.

## Ethics Statement

This review is a synthesis of previously published, publicly available scholarly articles and does not contain any original data. Consequently, the need for ethical approval from an institutional review board (IRB) or equivalent committee was considered not applicable. The authors confirm that all source material has been appropriately cited and referenced.

## Conflicts of Interest

**T.S. Higgins:** GlaxoSmithKline, Optinose, Regeneron Pharmaceuticals Inc., Sanofi—speaker and consultant; Medtronic, Stryker—consultant. **J.E. Douglas:** Sanofi—advisory board member. **R.C. Kern:** GlaxoSmithKline, Lyra, Regeneron Pharmaceuticals Inc., Sanofi—consultant. **J.N. Palmer:** Acclarent, Medtronic, Optinose—consultant; Sanofi—advisory board member. **S. Reitsma:** GlaxoSmithKline, Novartis, Sanofi—consultant and/or advisory board member, research funding. **M. Wagenmann:** ALK‐Abelló, AstraZeneca, GlaxoSmithKline, Novartis, Sanofi, Stallergenes Greer, Takeda—advisory board member, lecture fees, and research grants. **R. Goodman, A. Radwan:** Regeneron Pharmaceuticals Inc.—employees and shareholders. **M. Corbett, C. Almansa:** Sanofi—employees may hold stock and/or stock options in the company.

## Supporting information


**Video S1:** Importance of Smell Loss to Patients With Chronic Rhinosinusitis With Nasal Polyps: Options for Management and Recovery.

**Figure S1:** Scoring thresholds of commonly used psychophysical smell assessments. NA, not applicable; TDI, threshold, discrimination, and identification; UPSIT, University of Pennsylvania Smell Identification Test.

Supporting Information S1

## Data Availability

Data sharing not applicable to this article as no datasets were generated or analysed during the current study.
