# Supplementary material for: Importance of Smell Loss to Patients With Chronic Rhinosinusitis With Nasal Polyps: Options for Management and Recovery
Source: Clin Transl Allergy. 2026 Jan 31;16(2):e70149. doi: 10.1002/clt2.70149 (PMC12860424; doi:10.1002/clt2.70149)
Supplement: Supplementary file 2 — Figure S1: Scoring thresholds of commonly used psychophysical smell assessments. NA, not applicable; TDI, threshold, discrimination, and identification; UPSIT, University of Pennsylvania Smell Identification Test. [file CLT2-16-e70149-s001.docx]

**SUPPLEMENTARY FIGURE 1** Scoring thresholds of commonly used psychophysical smell assessments.

**
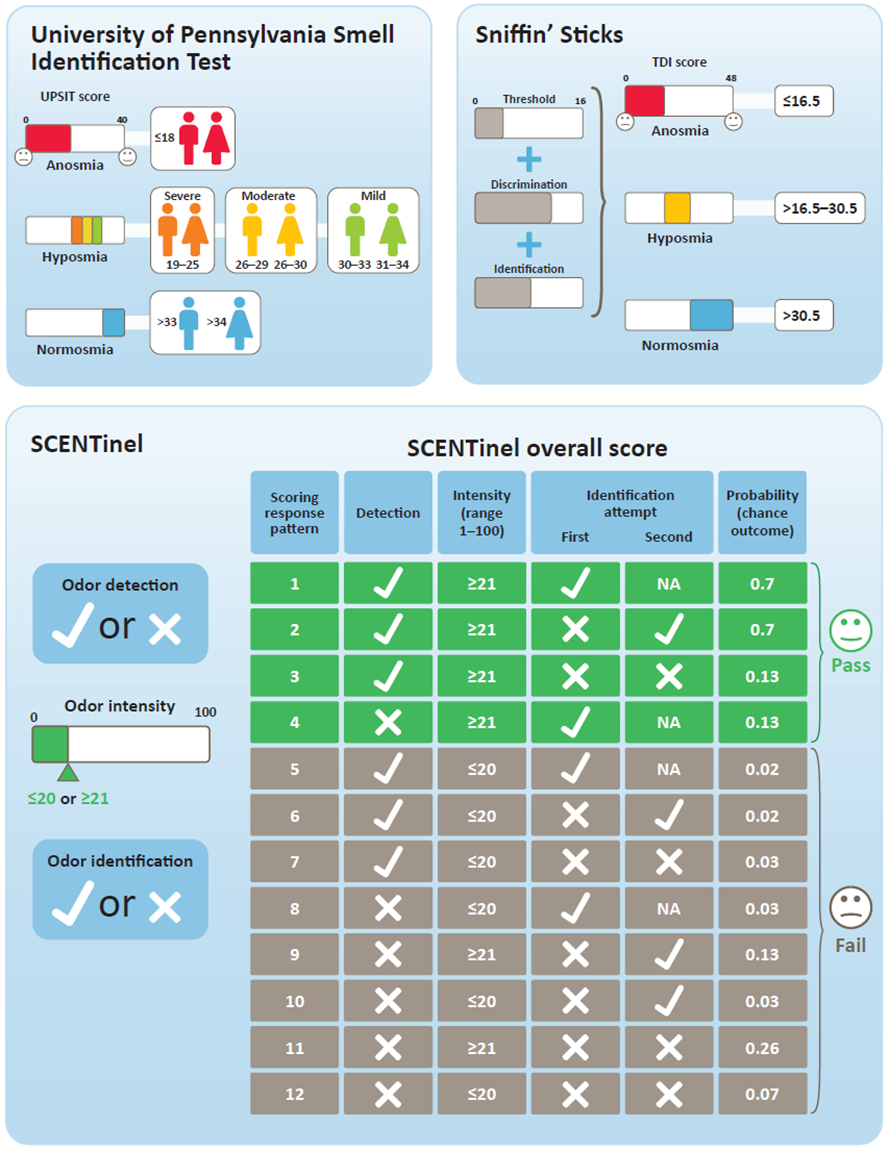
**

Abbreviations: NA, not applicable; TDI, threshold, discrimination, and identification; UPSIT, University of Pennsylvania Smell Identification Test.
