# Supplementary material for: Importance of Smell Loss to Patients With Chronic Rhinosinusitis With Nasal Polyps: Options for Management and Recovery
Source: Clin Transl Allergy. 2026 Jan 31;16(2):e70149. doi: 10.1002/clt2.70149 (PMC12860424; doi:10.1002/clt2.70149)
Supplement: Supplementary file 3 — Supporting Information S1 [file CLT2-16-e70149-s002.docx]

**Importance of smell loss to patients with chronic rhinosinusitis with nasal polyps:
Options for management and recovery**

VIDEO SCRIPT presented by Doctor Thomas Higgins

| **Frame (time)** | **Script** | **Visual elements** |
| --- | --- | --- |
| **1**  **(12 sec)** | Hello, I’m Doctor Thomas Higgins and I am pleased to introduce this review of the management and recovery of smell loss in patients with Chronic Rhinosinusitis with Nasal Polyps, or CRSwNP. | Talking head with title and affiliations on screen.  Doctor Thomas Higgins, MD, MSPH, MBA  Kentuckiana ENT, a Division of ENT Care Centers, Louisville, KY, USA |
| **2**  **(15 sec)** | CRSwNP is an inflammatory disease of the nasal cavity and paranasal sinuses.  Impaired sense of smell is one of the cardinal symptoms and can impact patients’ safety, mental health, and quality of life. | Cut-away of nasal cavity and paranasal sinuses, showing formation of nasal polyps.  Next to the graphic showing the disease, the words ‘Safety’, ‘Mental health’ and ‘Quality of life’ with a downward red arrow indicating worsening. |
| **4**  **(12 sec)** | Mechanisms of smell loss in CRSwNP are not well understood, but increasing evidence indicates the role of type 2 inflammatory mediators. | Include elements from Figure 1 graphic on potential mechanisms of smell loss. |
| **5**  **(12 sec)** | Management of smell loss in patients with CRSwNP typically involves steroid treatment.  But for patients whose disease remains uncontrolled, sinonasal surgery can remove the polyps. | Graphic from section 2, now showing a scalpel icon and the polyps disappearing. |
| **6**  **(20 sec)** | Surgery is effective in improving sense of smell but many patients experience the eventual recurrence of smell loss. | Elements indicating restored smell. Then a simple calendar graphic with the months passing, and a return of the loss of smell. |
| **7**  **(10 sec)** | Guidelines recommend biologics for some patients with CRSwNP, and data from clinical trials and real-world studies have demonstrated the effectiveness of biologics in improving sense of smell. | A syringe and antibody icons to indicate biologics, with a row of people to indicate testing in clinical trials.  A nose smelling an aroma with a tick icon to indicate positive results. |
| **8**  **(10 sec)** | Shared decision-making in the management of CRSwNP can help to identify treatments that are best suited to achieving patient goals. | Simple graphic of two people (a healthcare professional and a patient) having a conversation. |
| **9**  **(20 sec)** | In conclusion, reduced sense of smell is a burdensome symptom for patients with CRSwNP.  Maintaining improvements in sense of smell over the long-term can be challenging, and biologics may be recommended for some patients.  Given the impact of smell loss, shared decision-making is important in identifying the right treatment for each patient.  Thank you. | Talking head on screen. |
